# Indents

**Published:** 1918-12

**Authors:** 


### INDENTS

**☞Brief Articles of Technical or Scientific Value will receive notice and comment. Contributions invited.**

*Vulcanite Repair* Carbon tetra-chloride will cleanse the old vulcanite surfaces as well as chloroform and is not so expensive. Make a solution of the carbon chloride and vulcanite to a thick paste which should be smeared over the edges and scraped surfaces of the old vulcanite plate to be repaired. The vulcanite can then be packed over the painted joint or surface and when vulcanized a strong and durable repair will result.—Dr. Frahm, in *Pacific Gazette.*

*Oxide of Zinc and Oil of Cloves* It is not likely to be too frequently repeated that a temporary filling of oxide of zinc and oil of cloves, placed over softened dentine in a vital tooth will so dry out the dentine that it can be excavated with comfort to the patient. Such fillings may often remain in place for three months, and will then require a bur or sharp excavator to remove them. The influence of the filling is to reduce the inflammation of the pulp and the sensitiveness of the dentine.

Three months ago an adult case presented in which it seemed probable that the pulp must be removed from two vital teeth. There was no exposure, but the dentine over the area of the pulp was much softened. A temporary filling of oil of cloves and oxide of zinc was placed in each tooth and the patient dismissed. The patient recently returned to the office with the pulps vital and the teeth in fine condition. The temporary fillings have been removed, the dentine excavated without causing pain and the teeth filled.

The oxide of zinc and oil of cloves should be mixed to a very thick paste so that when it is patted with a spatula, moisture can just be seen. It should be patted to place in the cavity, and saliva immediately permitted to flow over it.—E. S. Ulsaver, in *Digest.*

*Present Status of the Treatment of Syphilis*

Dr. Jay F. Schamberg, Philadelphia: There are three recognized drugs in the treatment of syphilis—arsphenamin, mercury and the iodids. Arsphenamin is generally recognized to be the remedy of paramount value. Its effect on symptoms is due to the fact that it has a powerful destructive effect on the spirochete and that it may be administered in large dosage. Arsphenamin is fifty times less toxic for experimental animals than is mercury. Arsphenamin likewise has a roborant or tonic effect which mercury does not possess. The spirochete is vulnerable to arsphenamin and to mercury. The iodids do not appear to have direct destructive influence on the spirochete. Wechselmann has warned against the use of mercury precedent to the use of arsphenamin, on the grounds that most of the arsphenamin fatalities are due, in large part, to renal insufficiency, and that in the majority of cases it was mercury that damaged the kidneys. My belief is that the use of three inunctions of mercury a week is a valuable measure to associate with the use of arsphenamin, particularly in the primary and secondary stages of syphilis. No one is in an authoritative position today to state how long the treatment of syphilis should continue. Too often the physician will cease treatment after a single series of arsphenamin injections and perhaps a course of mercury, because the Wassermann reaction has become negative. Experience proves that such a course usually requires the later resumption of treatment, with valuable time lost. While it is difficult to prescribe any routine formula, we may at least indicate an irreducible minimum of treatment. Before any patient with syphilis is discharged from observation, a diagnostic spinal puncture should be made. —*Journal A. M. A.*

*Alkalis in the Treatment of Influenza*

It is universally agreed that in perverted metabolism by bacterial invasion, it is the acidosis that is fatal. When the system is saturated with alkalis, there is poor soil for bacterial growth. The baneful acids may be neutralized by harmless alkalis, and these seem to act almost specifically. I have uniformly employed, and always with good results, potassium citrate and sodium bicarbonate saturation by mouth, bowel and skin.

For the past three weeks, in the present influenza epidemic, I have seen an average of 100 private patients daily. There have been all degrees of frequency and severity, and in some houses as many as six patients.

As is well known in this epidemic, there has been a very high mortality in cases occurring in pregnant women. I have seen three instances of bronchopneumonia, or "lung patches," in pregnant women, two at term, with complete recovery.

Incidentally, I have made the use of the bed-pan imperative, and have absolutely refrained from administering any of the coal-tar series.

I explain to the afflicted persons that they must be patient, and that the fever, aches and pains are inevitable for a few days, and that they must be willing to forego the seductive relief afforded by acetylsalicylic acid (aspirin), acetanilid and other heart depressants. I employ cold applications and the ordinary stimulation by strychnin, aromatic spirits of ammonia, digitalis, quinine, etc. I have always withheld all depressants.

My very successful experience in this epidemic with the saturation of the system with harmless alkalis can not be dismissed as accidental or unique. It seems to represent an important new medical fact, or one apparently forgotten or generally overlooked. It is so simple and without any pssible objection that I urge its immediate trial empirically. Further investigation in laboratory and clinic may follow later.—Thomas C. Ely, M.D., in *Journal A. M. A.*

*Ulcerative Stomatitis* This disease is not present unless systemic vitality is greatly reduced by some general disease. It may follow the administration of mercury, when adequate prophylaxis of the teeth has not preceded the administration of the mercury.

The ulcers are usually located on the gum margins but may affect any part of the mucous membrane of the mouth. It is contagious and may affect a family and has become epidemic in soldiers' camps and in factories.

It is not always local, and symptoms characteristic of toxemia are frequently present. Intestinal symptoms, such as lack of appetite, temperature and depression may be present. The gangrenous ulcerations give off very offensive odors and the tissues become exceedingly sensitive and painful. The infection may spread to the periosteum and cause destruction of the alveolar process and loosening of the teeth.

The treatment is both local and systemic.

The teeth should first be thoroughly scaled and polished.

Temporary fillings should be inserted when needed.

Apply ten to twenty per cent. silver nitrate to the ulcerated areas.

Irrigate the mouth frequently with potassium per manganate solutions of proper strength.

Give patient rest, a non-irritating diet, and allow no alcohol or tobacco.

Give laxatives to secure competent elimination.

Administer internally potassium chlorate in solution of twenty-four grains to the ounce in teaspoonful doses every three hours. For children reduce the dose by making the solution of ten grains to the ounce of water.—Julio Endelman, in *Pacific Gazette.*

*What the Expert Anesthetist Should Be*

A specialist in any branch of medicine or surgery must have the foundation of a proper academic and professional education, with considerable general experience and a great deal of special study and practice of his chosen branch. Aside from this he must have a liking and aptitude for his specialty if he is to succeed in it. Furthermore, he must devote his time exclusively to his particular line of work. In anesthesia these requisites would comprehend a collegiate course, followed by four years at an A-1 medical school, which gives proper attention to anesthesia in its curriculum and clinics, an internship in a hospital in which anesthesia is under the control and supervision of experts, then post-graduate study in various centers where the latest methods of anesthesia may be studied and tried out, followed by intensive practice of the specialty in some chosen community in which there is an opportunity for becoming connected with several prominent surgical teams or hospitals in which surgery is on a distinct plane of excellence. Having achieved this much, the anesthetist owes it to himself to maintain an equipment of apparatus that will enable him to meet the anesthetic requirements for any surgical procedure with safety and comfort to the patient and satisfaction to the operator.—Dr. A. S. McCormick, in *Journal A. M. A.*

*Uniformed Dentists' Assistants*

A writer in the *Dental Record* takes exceptions to what he calls the *parade* of the gowned trained nurse in the dental office. There may be an excuse for such an assistant to help calm nervous patients or to lend assistance to the operator and to help facilitate his work. He, however, objects to officiousness and the irritating chatter of the nurse often not in tune with the harmony of the "buzzer"—dental engine. He thinks the proper sphere of the garbed lady assistant is out of the sight of the patient, quietly busy with the sterilization of the instruments and other unobtrusive duties that are necessary to the orderly conduct of the office business. She has no good reason as an entertainer or show piece in a properly conducted dental office, whether uniformed or in mufti.

### UNCLE SAM'S BOYS

From the wilds of far Alaska
  To the Caribbean Sea,—
From Hawaii, Porto Rico,
  Come these sons of Liberty.
In the North-land, in the South-land,
  East and West—they hear the call,
And with firm, united purpose
  They are hast'ning, one and all,
For the hot, red blood is throbbing
  In each young, impatient heart,
And in Freedom's mighty struggle
  They are longing to take part.
Starred upon the Roll of Honor
  From all corners of the world,
Foreign names and foreign faces
  Stand with Stars and Stripes unfurled.
Czech and Slovak, Serb, Armenian,
  Swede, Norwegian, Pole and Greek,
Magyar, Finn—what matter forebears
  When one common goal they seek?—
When for Justice, Mercy, Freedom,
  Side by side they join the fray,
Uncle Sam is proud to call them,
  Each and all,—his boys today!

—E. E. Brown.

*The Telephone in Surgery* The telephone is being put to many and strange uses these days, especially in connection with the war. Among them perhaps none is stranger than its use in surgery. The army surgeon now finds that by connecting one terminal of a telephone with a moistened electrode applied to the patient's skin and the other terminal to a metallic probe it is possible to discover a bullet located in the man's body. Directly the probe touches the bullet imbedded in the tissue a grating sound is heard in the telephone receiver. This method not only reduces the time of search but prevents serious disturbance of the patient's tissues.—*Digest.*

